# Ticks are more suitable than red foxes for monitoring zoonotic tick-borne pathogens in northeastern Italy

**DOI:** 10.1186/s13071-018-2726-7

**Published:** 2018-03-20

**Authors:** Graziana Da Rold, Silvia Ravagnan, Fabio Soppelsa, Elena Porcellato, Mauro Soppelsa, Federica Obber, Carlo Vittorio Citterio, Sara Carlin, Patrizia Danesi, Fabrizio Montarsi, Gioia Capelli

**Affiliations:** 1 Istituto Zooprofilattico Sperimentale delle Venezie, Legnaro, Italy; 2 Local Health Unit, ULSS1-Dolomiti, Belluno, Italy

**Keywords:** Tick-borne pathogens, *Ixodes ricinus*, Red fox, Zoonosis, Monitoring

## Abstract

**Background:**

Northeastern Italy is a hotspot for several tick-borne pathogens, transmitted to animals and humans mainly by *Ixodes ricinus*. Here we compare the results of molecular monitoring of ticks and zoonotic TBPs over a six-year period, with the monitoring of red foxes (*Vulpes vulpes*) in an endemic area.

**Results:**

In the period 2011–2016, 2,578 ticks were collected in 38 sites of 20 municipalities of Belluno Province. Individual adults (264), pooled larvae (*n* = 330) and nymphs (*n* = 1984) were screened for tick-borne encephalitis virus, *Borrelia burgdorferi* (*s*.*l*.), *Rickettsia* spp., *Babesia* spp., *Anaplasma phagocytophilum* and “*Candidatus* Neoehrlichia mikurensis” by specific SYBR green real-time PCR assays and sequencing. The spleens of 97 foxes, culled in the period 2015–2017 during sport hunting or population control programs, were also screened. Overall, nine different pathogens were found in *I*. *ricinus* nymph and adult ticks: *Rickettsia helvetica* (3.69%); *R*. *monacensis* (0.49%); four species of the *B*. *burgdorferi* (*s*.*l*.) complex [*B*. *afzelii* (1.51%); *B*. *burgdorferi* (*s*.*s*.) (1.25%); *B*. *garinii* (0.18%); and *B*. *valaisiana* (0.18%)]; *A*. *phagocytophilum* (3.29%); “*Candidatus* N. mikurensis” (1.73%); and *Babesia venatorum* (0.04%). Larvae were collected and screened in the first year only and two pools (0.6%) were positive for *R*. *helvetica*. Tick-borne encephalitis virus was not found in ticks although human cases do occur in the area. The rate of infection in ticks varied widely according to tick developmental stage, site and year of collection. As expected, adults were the most infected, with 27.6% harboring at least one pathogen compared to 7.3% of nymphs. Pathogens with a minimum infection rate above 1% were recorded every year. None of the pathogens found in ticks were detectable in the foxes, 52 (54%) of which were instead positive for *Babesia* cf. *microti* (also referred to as *Babesia microti*-like, “Theileria annae”, “Babesia annae” and “Babesia vulpes”).

**Conclusions:**

The results show that foxes cannot be used as sentinel animals to monitor tick-borne pathogens in the specific epidemiological context of northeastern Italy. The high prevalence of *Babesia* cf. *microti* in foxes and its absence in ticks strongly suggests that *I*. *ricinus* is not the vector of this pathogen.

**Electronic supplementary material:**

The online version of this article (10.1186/s13071-018-2726-7) contains supplementary material, which is available to authorized users.

## Background

The territory of northeastern Italy is occupied mainly by Alpine and pre-Alpine areas, characterized by a well-conserved biocenosis including rodents, carnivores, deer, wild boars and birds. The humid climate and availability of hosts provide favorable conditions for the proliferation of *Ixodes ricinus*, the most abundant tick in the area [1], and for the survival and maintenance of tick-borne pathogen (TBP) life-cycles.

The TBP *Borrelia burgdorferi* was first isolated from *I*. *ricinus* in northeastern Italy in 1989 [2]. Several other pathogens were later discovered in *I*. *ricinus* questing ticks, i.e. *B*. *afzelii*, *B*. *garinii*, *B*. *burgdorferi* (*s*.*s*.), *B*. *valaisiana*, *B*. *lusitaniae*, *Rickettsia helvetica*, *R*. *monacensis*, *R*. *raoultii*, *R*. *limoniae*, “*Candidatus* Neoehrlichia mikurensis”, *Anaplasma phagocytophilum*, tick-borne encephalitis flavivirus, *Babesia venatorum*, *Ba*. *capreoli* and *Ba*. *microti-*like [1, 3–20]. The northeast also accounts for the majority of human cases of Lyme borreliosis and tick-borne encephalitis in Italy [21].

In the area of our survey, Belluno Province, the first European sequence of the bacterium (later suggested to be “*Ca.* N. mikurensis”) was found in *I*. *ricinus* detached from humans [22, 23] and then in questing ticks [24, 25].

As a result, surveillance programmes for tick-borne infections have been implemented locally, often following the upsurge of human cases. They generally aim to (i) assess the infection rate in ticks; (ii) monitor variations of pathogen prevalence; and (iii) detect the introduction of any new pathogens or vectors. The programmes are mainly based on the collection and molecular screening of *I*. *ricinus* ticks, along with occasional serological surveys on domestic animals or forestry workers [26]. The collection of ticks and their molecular screening is, however, time-consuming and costly. For example, the costs for a survey conducted during 2006–2008 in northeastern Italy, including travel expenses, staff, molecular analysis and sequencing, was estimated at over €20,000 per year [19].

Alternatively, wild mammals that host *I*. *ricinus* ticks and are susceptible to TBPs may be used as sentinels. The survey area hosts 42 mammalian species including deer (chamois, red deer, roe deer, mouflon), bats, foxes, marmots, badgers, stoats, martens, squirrels, lynxes, wolves, in addition to bears and, since 2014, wildcats [27]. The red fox (*Vulpes vulpes*) could be a good candidate for surveillance since it is widespread, abundant [28], and subject to sport hunting and possible population control plans. In previous studies, blood or spleen samples from red foxes were found positive for TBPs transmitted by *Ixodes* spp. such as *A*. *phagocytophilum*, with a prevalence ranging between 0.6–16.6% in Italy [29] and other European countries [30–33], and *B*. *burgdorferi*, found in 1.42% of foxes in Romania [31].

Being the main reservoir of important zoonotic pathogens (*Trichinella britovi*, *Echinococcus multilocularis*, rabies virus), surveillance programs on red foxes are already in place in our study area, making sampling easier and more cost-effective.

The aim of this study was to assess the suitability of red foxes, assigned to Istituto Zooprofilattico Sperimentale delle Venezie, as sentinel animals for zoonotic TBPs, with the molecular screening of *I*. *ricinus*, in northeastern Italy.

## Methods

### Study area and sampling

Between 2011 and 2016, ticks were collected by forest rangers and local health unit personnel, by standard dragging using a 1 m^2^ white flannel cloth. Sampling was performed monthly in five sites during the peak of *Ixodes* spp. activity, i.e. in spring (April, May and June) and autumn (September and October), based on previous experience in northeastern Italy [19, 34]. A further 33 sites were visited only sporadically, from one to three times. The altitude of the sampling sites ranged from 340 to 1,792 meters above sea level (masl).

**Fig. 1:**
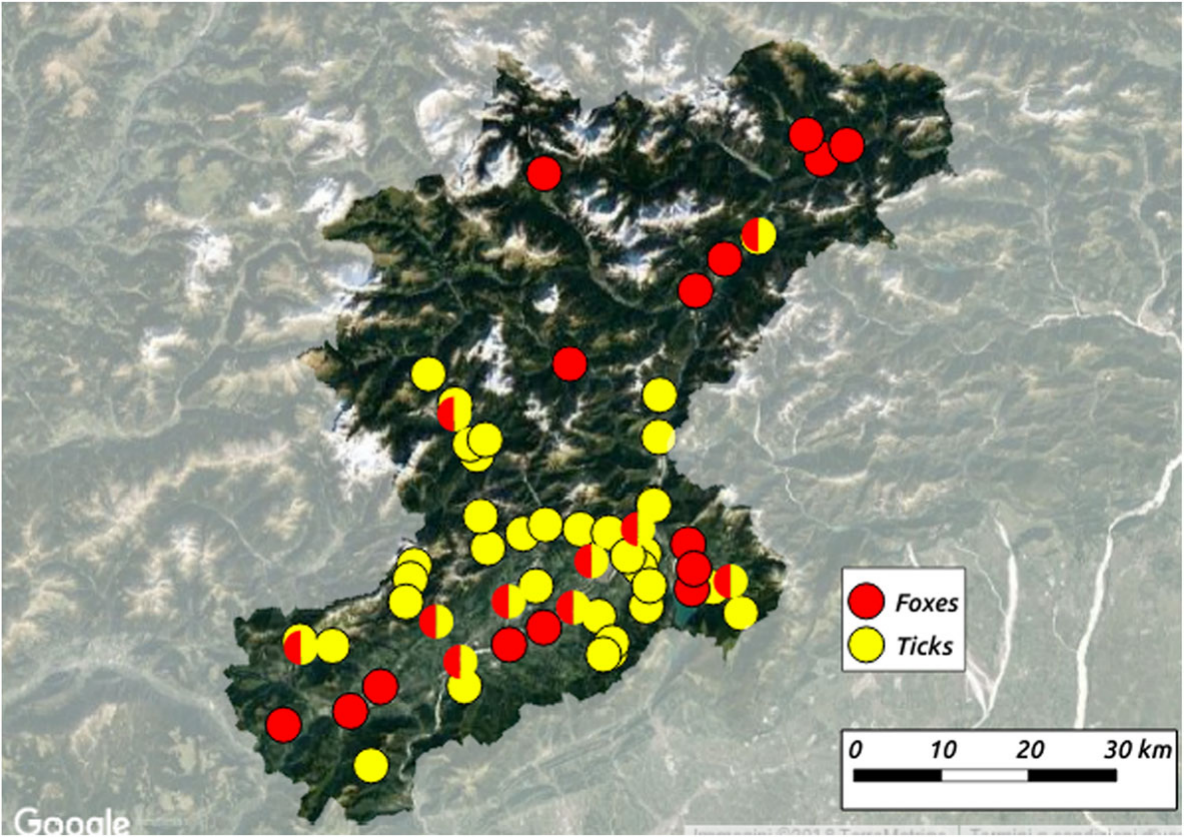
Municipalities where *Ixodes ricinus* ticks and red foxes (*Vulpes vulpes*) were sampled in the Belluno Province, northeastern Italy. Mixed circles (yellow/red) represent municipalities where both ticks and foxes were sampled

All 38 sampling sites were located in 20 municipalities within the Dolomiti Bellunesi National Park in the Province of Belluno (3600 km^2^), an area of the Veneto Region neighboring the Friuli Venezia Giulia and Trentino Alto Adige regions (Italy), and Austria (Fig. 1). The climate is sub-continental, with cold and often snowy winters and mild, warm summers. Belluno Province is humid, rich in water, and crossed by the wide Piave River. The average annual temperature is 9 °C, and the average annual precipitation is above 1300 mm.

Once collected, the ticks were killed by freezing, counted, grouped according to their developmental stage, and identified according to morphological features [35, 36]. They were then stored at -80 °C until molecular analysis.

From November 2015 to January 2017, a spleen sample was also collected from red foxes (*V*. *vulpes*) culled during sport hunting or population control programs and assigned to Istituto Zooprofilattico Sperimentale delle Venezie for the surveillance of zoonotic agents. Spleens were collected in 25 municipalities of the same province, with an altitude ranging from 263 to 1229 masl (Fig. 1), and were kept frozen at -20 °C until testing.

### Molecular analysis

Nucleic acids were extracted from the spleen of each fox, from adult ticks, pooled larvae (maximum 20 specimens) and pooled nymphs (maximum 10 specimens). Larvae were collected and screened for TBPs in 2011 only, due to their low infection rates and to budget optimization. In the following years, only 15 larvae detached from collectors were included in the study.

DNA was extracted from *I*. *ricinus* tick samples using the All Prep DNA/RNA mini Kit (Qiagen, Valencia, CA, USA), according to the manufacturer’s instructions, and then kept frozen at -80 °C. DNA was extracted from spleen samples using DNeasy Blood & Tissue Kit (Qiagen), according to the manufacturer’s instructions, and stored at a temperature of -20 °C.

DNA from tick and spleen samples was amplified by conventional PCR or SYBR Green real-time PCR (rPCR) assays for *Borrelia burgdorferi* (*s*.*l*.), *Rickettsia* spp., *Babesia* spp., *A*. *phagocytophilum* and “*Ca.* N. mikurensis”. The target genes, primers used and related references are listed in Table 1.

**Table 1:** Target pathogens, molecular method, target genes and primers used in this study

Target	Method	Gene	Primer	Amplicon size (bp)	Reference
*B*. *burgdorferi* (*s*.*l*.)	Traditional PCR	*Flagellin*	FLA1; FLA2	482	[51]
“*Candidatus* N. mikurensis”	Traditional PCR	*groEL*	NM-350s (5'-GTG TAA TGA CAA AGT TGG TGA TGG-3'); NM-1152as	802	This study; [52]
*A*. *phagocytophilum*	SYBR green rPCR	*msp2*	msp2-3f ; msp2-3r	334	[53]
*Rickettsia* spp.	SYBR green rPCR	*OmpB*	rompB OFm; rompB ORm	489	[54]
*Babesia* spp.	SYBR green rPCR	*18S* rRNA	BJ1; BN2	411–452	[55]
DNA extraction control	Traditional PCR	*18S* rRNA	18SU; 18SD	488	[37]
TBE virus	TaqMan rRT-PCR	3' non-coding region	F-TBE 1; R-TBE 1 TBE-Probe-WT	67	[38]
RNA extraction control	TaqMan rRT-PCR	*16S* rRNA	F-16sIxodes; R-16sIxodes; 16s-Ixodes-Probe	97	[38]

To ensure the effectiveness of DNA extraction, a PCR was applied targeting the *18S* rRNA gene internal control (Table 1) [37]. Negative (sterile water) and positive controls (DNA of *B*. *burgdorferi* (*s*.*s*.), *R*. *helvetica*, *Ba*. *venatorum*, *A*. *phagocytophilum* and “*Ca.* N. mikurensis”) were included in each run.

PCR products were sequenced, in both directions, using the Big Dye Terminator v.3.1 cycle sequencing kit (Applied Biosystems, Foster City, CA, USA). The products of the sequencing reactions were purified using PERFORMA DTR Ultra 96-Well kit (Edge BioSystems, Gaithersburg, MD, USA), and sequenced in a 16-capillary ABI PRISM 3130xl Genetic Analyzer (Applied Biosystems). Sequence data were assembled and edited with SeqScape software v2.5 (Applied Biosystems). The resulting sequences were aligned and compared with representative sequences available in GenBank.

RNA from tick samples was amplified by a specific real-time PCR (rRT-PCR) for TBE virus detection, as described elsewhere [38]. To ensure the effectiveness of RNA extraction, a real-time PCR targeting the *16S* rRNA gene of *Ixodes* spp. was applied [38] (Table 1).

### Statistical analysis

For individual samples (adult ticks and foxes), the infection rate (IR) was calculated as the number of positive ticks/examined specimens. For pooled samples, the IR was calculated as the number of positive pools/total ticks examined in the pools (i.e. the minimum infection rate; https://tinyurl.com/y8uuopc6). Co-infections could not be estimated for pooled samples.

The significance of IR differences according to developmental stage and year of collection was tested using the Chi-square test (*χ*^2^) or Fisher’s exact test, where appropriate.

Data and tests were managed by SPSS software for Windows, v.13.0 (SPSS Inc., Chicago, IL, USA). Maps were produced using Qgis 2.14.18-Essen (2017, http,//https://qgis.org/it/site//) and graphs drawn up using Tableau desktop v.10.4.0 Professional Edition© 2017 (Tableau Software Inc., Seattle, WA, USA).

## Results

In the six years of monitoring, 2578 *Ixodes ricinus* ticks were collected and screened for TBPs. No other tick species were found in the study area. A total of 565 DNA/RNA extracts were obtained from 264 adults, 24 pools of larvae (*n* = 330), and 277 pools of nymphs (*n* = 1984) (see Additional file 1: Table S1).

Overall, nine different pathogens were found to be circulating in the province, vectored by *I*. *ricinus* nymphs and adults: *Rickettsia helvetica* (3.69%); *R*. *monacensis* (0.49%); four species of *Borrelia burgdorferi* (*s*.*l*.) [*B*. *afzelii* (1.51%); *B*. *burgdorferi* (*s*.*s*.) (1.25%); *B*. *garinii* (0.18%); and *B*. *valaisiana* (0.18%)]; *Anaplasma phagocytophilum* (3.29%), “*Ca.* Neoehrlichia mikurensis” (1.73%), and *Babesia venatorum* (0.04%) (Table 2). Larvae were collected and screened in the first year only (*n* = 315) and two pools (0.6%) were positive for *R*. *helvetica*. In the following years, 15 larvae were detached by forest rangers during sampling and two were found positive for *R*. *helvetica* and “*Ca.* mikurensis”, respectively. Tick-borne encephalitis virus was not found in ticks even though human cases do regularly occur in the area.

**Table 2:** Species and infection rates (%) of pathogens found in 2248 *Ixodes ricinus* nymphs and adults collected from 2011 to 2016, and sites positive for each pathogen by year of collection

Species	No. of infected ticks	%	95% CI	Positive sites (*n* = total sites monitored)					
				2011 (*n* = 5)	2012 (*n* = 13)	2013 (*n* = 14)	2014 (*n* = 14)	2015 (*n* = 12)	2016 (*n* = 8)
*Rickettsia* spp.	91	4.05	3.23–4.86	2	7	6	6	4	5
*Rickettsia helvetica*	83	3.69	2.91–4.47	2	6	6	4	4	5
*Anaplasma phagocytophilum*	74	3.29	2.55–4.03	2	4	4	5	4	1
*Borrelia burgdorferi* (*s*.*l*.)	70	3.11	2.40–3.83	4	3	5	1	7	5
“*Ca.* Neoehrlichia mikurensis”	39	1.73	1.20–2.27	4	3	3	4	3	–
*Borrelia afzelii*	34	1.51	1.01–2.02	2	3	5	–	6	4
*Borrelia burgdorferi* (*s*.*s*.)	28	1.25	0.79–1.70	3	1	–	–	–	–
*Rickettsia monacensis*	11	0.49	0.20–0.78	1	3	–	3	–	1
*Borrelia garinii*	4	0.18	0.004–0.352	1	–	–	–	2	–
*Borrelia valaisiana*	4	0.18	0.004–0.352	–	–	–	1	1	2
*Babesia venatorum*	1	0.04	0.000–0.132	–	–	–	–	–	1

The rate of infection in ticks varied widely according to tick developmental stage, site and year of collection (Table 3, Additional file 2: Figure S1 and Additional file 3: Figure S2). As expected, adults were more infected, with 27.6% harboring at least one pathogen, followed by nymphs (7.3%) (*χ*^2^ = 109.780, *df* = 1, *P* = 0.0001). Four adults (0.7%) were co-infected with two pathogens each, i.e. one tick harbored *B*. *burgdorferi* (*s*.*s*.) + *A*. *phagocytophilum*, one tick *R*. *helvetica* + *B*. *afzelii*, one tick *R*. *helvetica* + *A*. *phagocytophilum* and one tick *R*. *monacensis* + *A*. *phagocytophilum*.

**Table 3:** Number of nymphs and adult ticks collected and infection rates (%) of tick-borne pathogens according to developmental stage and year of collection

Year	Nymphs		Adults		Total	
	positive/tested	% (95% CI)	positive/ tested	% (95% CI)	positive/ tested	% (95% CI)
2011	39/475	8.2 (5.74–10.6)	33/70	47.1 (35.45–58.84)	71/545	13.0 (10.20–15.85)
2012	27/443	6.1 (3.87–8.32)	17/80	21.3 (12.29–30.21)	44/523	8.4 (6.03–10.79)
2013	18/292	6.2 (3.41–8.92)	7/40	17.5 (5.72–29.28)	25/332	7.5 (4.69–10.37)
2014	17/201	8.5 (4.61–12.30)	10/34	29.4 (14.10–44.73)	27/2354	11.5 (7.41–15.57)
2015	28/330	8.5 (5.48–11.49)	2/17	11.8 (0.00–27.08)	30/347	8.6 (5.69–11.60)
2016	16/243	6.6 (3.47–9.70)	4/23	17.4 (1.90–32.88)	20/266	7.5 (4.35–10.69)
Total	145/1984	7.3 (6.16–8.45)	73/264	27.6 (22.26–33.05)	218/2248	9.7 (8.47–10.92)

Pathogens with an IR above 1% were recorded every year (Table 2). *Borrelia burgdorferi* (*s*.*s*.) was detected only in 2011 and 2012, *B*. *garinii* in 2011 and 2015, *B*. *valaisiana* from 2014 to 2016, and *Ba*. *venatorum* only in 2016.

The overall IR in nymphs was stable over the years, ranging between 6.1–8.5% (*χ*^2^ = 3.329, *df* = 1, *P* = 0.6494), while adults showed large IR variability, with a significantly higher IR in 2011 (47.1%; *χ*^2^ = 20.4, *df* = 5, *P* = 0.0010) compared to the following years, when the IR ranged, but not significantly so (*χ*^2^ = 2.813, *df* = 4, *P* = 0.5896), from 11.8 to 29.4% (Table 3).

Specifically, in the five permanent sites the IRs in nymphs and adult ticks ranged as follows by species or pathogen complexes: *Rickettsia* spp. between 2.0–6.3%; *B*. *burgdorferi* (*s*.*l*.) between 0.4–4.9%, *A*. *phagocytophilum* between 0.4–6.1%, and “*Ca.* N. mikurensis” between 0–3.3% (Additional file 2: Figure S1).

The pattern of IRs of TBPs across the years, in nymphs and adults, in the five permanent sites varied greatly (Additional file 3: Figure S2) among the different sites and also within the same site.

Concerning fox samples, a total of 97 foxes culled in Belluno Province were assigned to our laboratories: 70 from November 2015 to March 2016 and 27 from September 2016 to January 2017. None of the pathogens searched for in ticks was found in the foxes, while 52 foxes (54%) tested positive for *Babesia* cf. *microti* (syns. *Babesia microti*-like, “Theileria annae”, “Babesia annae”, “Babesia vulpes”). The sequences obtained from all 52 positive samples were identical to each other. Three representative sequences (one per year) were submitted to GenBank (accession numbers MG451837-MG451839). The IR rate was similar for foxes assigned in the periods 2015–2016 and 2016–2017 (50 *vs* 63%) (*χ*^2^ = 0.847, *df* = 1, *P* = 0.3574).

**Fig. 2:**
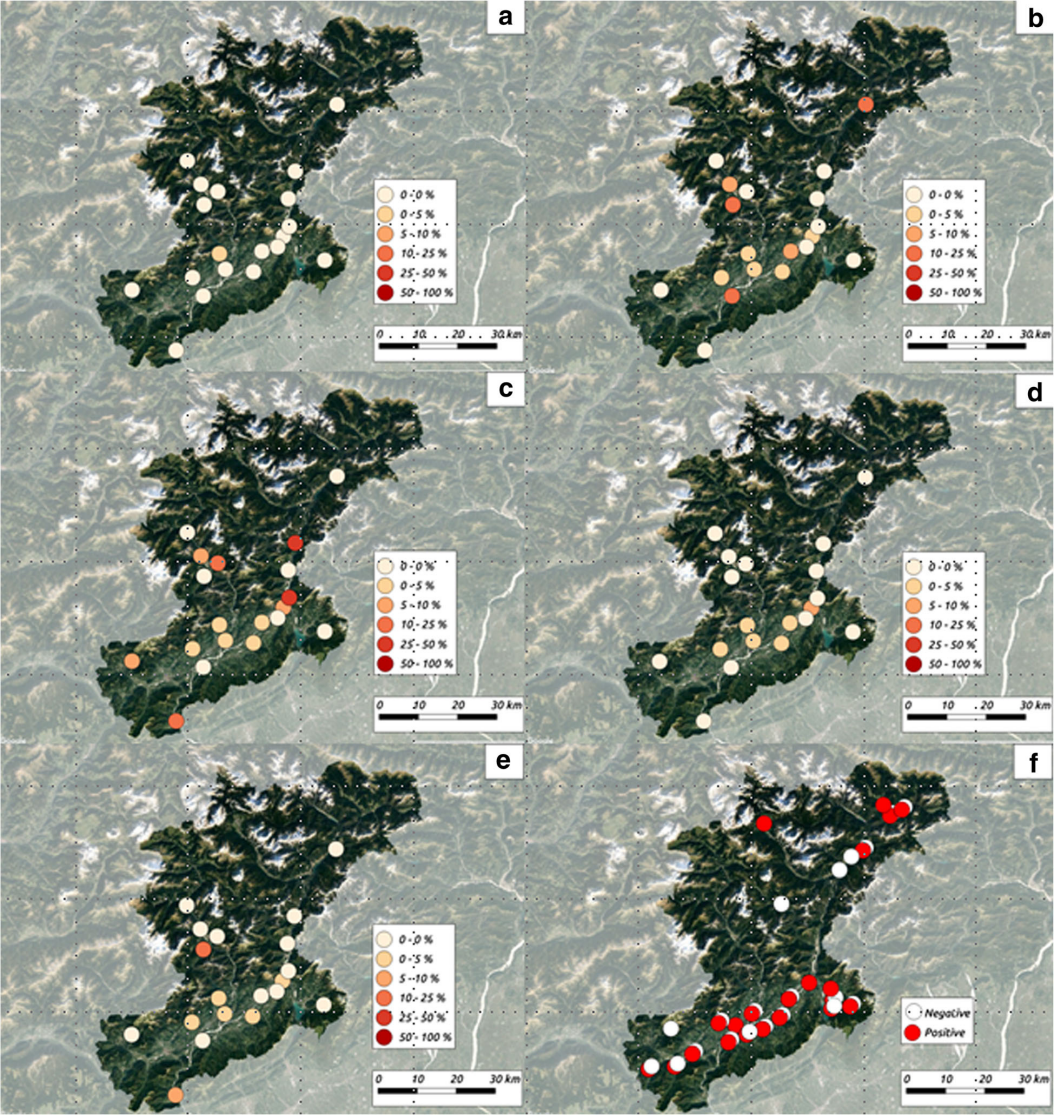
Positive municipalities and range of infection rates for tick-borne pathogens found in ticks (**a**-**e**) and red foxes (**f**). **a** *Babesia venatorum*, **b** *Borrelia burgdorferi* complex, **c** *Rickettsia* spp., **d** *Anaplasma phagocytophilum*, **e** “*Candidatus* Neoehrlichia mikurensis”, **f** *Babesia* cf. *microti*

*Babesia* cf. *microti* was found in all but four municipalities (Fig. 2).

## Discussion

The results of this study confirmed that annual monitoring of *Ixodes ricinus* is a good surveillance method for zoonotic TBPs in the Province of Belluno. Protracted monitoring also provided information on rare pathogens and highlighted spatial-temporal fluctuations in tick populations, testifying to the focal nature of TBP life-cycles, which are in turn linked to variations in reservoir density and amplifying hosts [39]. Tick-borne encephalitis virus, known to affect humans in the province [25], remained undetected. This is not surprising as tick-borne encephalitis virus is restricted to distinct, sometimes very small foci all over Europe [40]. For this virus, notification of human cases and serological surveys on both domestic grazing animals and population groups at risk of infection remain the most informative systems [41].

Our results also showed that monitoring zoonotic TBPs in the specific context of northeastern Italy cannot be based on the currently available foxes as sentinel animals.

The infection rate of TBPs in red foxes is correlated with tick species and abundance in the environment, and with the infection rate of pathogens in ticks [30, 32, 42]. In previous European studies, blood or spleen samples of the red fox were found positive for several TBPs, namely *A*. *phagocytophilum*, *B*. *canis*, *Bartonella rochalimae*, *B*. *burgdorferi*, “*Ca.* Neoehrlichia sp.”, *Coxiella burnetii*, *Hepatozoon canis* and *Ehrlichia canis* (Table 4).

**Table 4:** Tick-borne pathogen species and their prevalence (%) in red foxes (*Vulpes vulpes*) in European countries

Pathogens	Foxes tested	%	Country	Reference
*A*. *phagocytophilum*	506	0.6	Austria	[33]
	25	4.0	Czech Republic	[56]
	415	12.5	Hungary	[30]
	122	8.2	Germany	[43]
	150	16.6	Italy	[29]
	153	0.65	Italy	[32]
	353	2.5	Romania	[31]
	162	3.0	Switzerland	[57]
*Babesia* cf. *microti*^a^	36	50.0	Austria	[58]
	351–506	50.7–25.7^b^	Austria	[33]
	191	5.0	Croatia	[59]
	121	46.5	Germany	[48]
	195	47.5	Germany	[60]
	316	14.6	Great Britain	[61]
	404	20.0	Hungary	[62]
	153	22.9	Italy	[32]
	78	37.2	Italy	[63]
	180	59.0	Italy	[64]
	91	69.2	Portugal	[65]
	300	9.7	Slovakia	[66]
*Babesia canis*	351	0.3	Austria	[33]
	91	1.1	Portugal	[65]
*Bartonella rochalimae*	506	0.2	Austria	[33]
*Borrelia burgdorferi* (*s*.*l*.)	353	1.4	Romania	[31]
“*Ca.* Neoehrlichia sp.”	164	0.6	Austria	[67]
	506	0.4	Austria	[33]
*Coxiella burnetii*	153	1.96	Italy	[32]
*Ehrlichia canis*	105	52.0	Italy	[68]
	153	44.4	Italy	[32]
	13	31.0	Italy	[68]
	180	6.1	Italy	[64]
*Hepatozoon canis*	36	58.3	Austria	[58]
	351–506	18.5–29.8^b^	Austria	[33]
	191	23.0	Croatia	[59]
	415	22.2	Hungary	[30]
	153	49.0	Italy	[32]
	78	53.8	Italy	[63]
	119	13.4	Italy	[69]
*Rickettsia helvetica*	162	1.0	Switzerland	[57]
*Rickettsia* spp.	180	5.0	Italy	[64]

^a^Syns. *B*. *microti-*like, “Theileria annae”, “B. annae”, “B. vulpes”

^b^In blood and spleen of foxes, respectively

Variable prevalences of *A*. *phagocytophilum* have been found in foxes around Europe, especially in central eastern European countries [30, 31, 43], where *I*. *ricinus* meets all the criteria to be a very efficient vector [44]. Interestingly, two surveys on TBPs in foxes performed eight years apart in the same province in central Italy, produced contrasting results for the prevalence of *A*. *phagocytophilum*, which fell from 16.6% in 2007/2008 [29] to 0.65% in 2015/2016 [32]. Between November 2013 and March 2015, a similar low prevalence (0.6%) was also found in Austria [33], a country bordering northern Italy. Nevertheless it is difficult to interpret these results due to the paucity of data on fox abundance and density. In the case of Belluno Province, we could infer a maximum possible TBP prevalence of 3.03% in foxes, based on 97 sampled animals testing negative and on an estimated density of 3.38 foxes/km^2^ [45]. However, this estimate of abundance can vary during the year according to fox ecology and can differ even on a small geographical scale, depending on different factors, such as food availability. In our case, a density of 3.38 foxes/km^2^ was estimated in the southern countryside of Belluno Province, and could be misleading when considering the Alpine part of the territory.

Although a higher sample size would have increased the chance of finding positive foxes, the implementation of fox sampling was out of the scope of our study, which was to search for an alternative, low cost system of monitoring zoonotic TBPs, considering the current numbers of animals already sent to our laboratories.

The only TBP detected in the foxes examined here was *Babesia* cf. *microti*. More than 50% of foxes harbored this protozoan, in keeping with other European countries where prevalences of up to 69% have been reported (Table 4). The high prevalence of *Babesia* cf. *microti* in foxes and its absence in ticks strongly suggest that *I*. *ricinus* is not the vector of this pathogen. Accordingly, this protozoan is also present in countries where *I*. *ricinus* is absent, such as North America and Israel [46]. *Ixodes hexagonus* has been claimed to be a possible vector [47] and DNA of *B*. *microti*-like has been detected in all of the most common ticks infesting foxes in continental Europe [28], i.e. *I. hexagonus*, *I. ricinus *[48, 49], *I. canisuga* [48] and *Dermacentor reticulatus* [50]. The presence of nucleic acids of pathogens in hematophagous arthropods is, however, a common finding and may not be related to their vectorial status. Other mechanisms of transmission are likely involved in the maintenance in nature of *Babesia* cf. *microti*, e.g. through the ingestion of infected ticks, as in the case of *H*. *canis*, another protozoan found at high prevalence in foxes (Table 4), or by vertical transmission, as for other *Babesia* species [46].

## Conclusions

In areas endemic for vector-borne diseases, surveillance programmes are implemented to detect pathogens and define their spread. In the specific epidemiological context of northeastern Italy, and with the exception of tick-borne encephalitis virus, the molecular screening of TBPs in vector ticks remains a more efficient system than the screening of foxes as sentinel animals. The screening of foxes confirmed instead that *Babesia* cf. *microti* is endemic in northern Italy, as in many other European countries. Identification of the tick species vectoring the pathogen and the presence of alternative mechanisms of transmission are the next research tasks to be conducted on this protozoan.

## Additional files

Additional file 1: **Table S1.** Ticks collected in 2011–2016 organized in pools, data of sites and results of molecular screening. (XLS 172 kb)

Additional file 2: **Figure S1.** Overall infection rates (IR) of tick-borne pathogens found in *Ixodes ricinus* ticks in the 5 permanent sites monitored in 2011–2016. *Abbreviations*: Apha, *Anaplasma phagocytophilum*; Bbs.l., *Borrelia burgdorferi* (*sensu lato*) complex; Bafz, *Borrelia afzelii*; Bbs.s., *Borrelia burgdorferi* (*sensu stricto*); Bval, *Borrelia valaisiana*; CNmi, “*Candidatus* Neoehrlichia mikurensis”; Rhel, *Richettsia helvetica*; Rmon, *Rickettsia monacensis*. (TIFF 29 kb)

Additional file 3: **Figure S2.** Pattern of tick-borne pathogens found in *Ixodes ricinus* in the five permanent sites according to year of sampling. *Abbreviations*: Apha, *Anaplasma pagocytophilum*; Bbs.l., *Borrelia burgdorferi* (*sensu lato*) complex; Bafz, *Borrelia afzelii*; Bbs.s., *Borrelia burgdorferi* (*sensu stricto*); Bval, *Borrelia valaisiana*; CNmi, “*Candidatus* Neoehrlichia mikurensis”; Rhel, *Richettsia helvetica*; Rmon, *Rickettsia monacensis*. (TIFF 161 kb)

## Abbreviations

masl: meters above sea level
IR: infection rate
TBP: tick-borne pathogen
PCR: polymerase chain reaction
