# Supplementary figures and images for: Ticks are more suitable than red foxes for monitoring zoonotic tick-borne pathogens in northeastern Italy
Source: Parasit Vectors. 2018 Mar 20;11:137. doi: 10.1186/s13071-018-2726-7 (PMC5859681; doi:10.1186/s13071-018-2726-7)

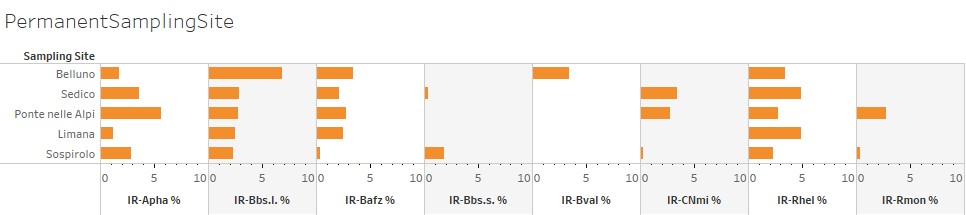

Supplement: Supplementary file 2 — Figure S1. Overall infection rates (IR) of tick-borne pathogens found in Ixodes ricinus ticks in the 5 permanent sites monitored in 2011–2016. Abbreviations: Apha, Anaplasma phagocytophilum; Bbs.l., Borrelia burgdorferi (sensu lato) complex; Bafz, Borrelia afzelii; Bbs.s., Borrelia burgdorferi (sensu stricto); Bval, Borrelia valaisiana; CNmi, “Candidatus Neoehrlichia mikurensis”; Rhel, Richettsia helvetica; Rmon, Rickettsia monacensis. (TIFF 29 kb) [file 13071_2018_2726_MOESM2_ESM.tif]

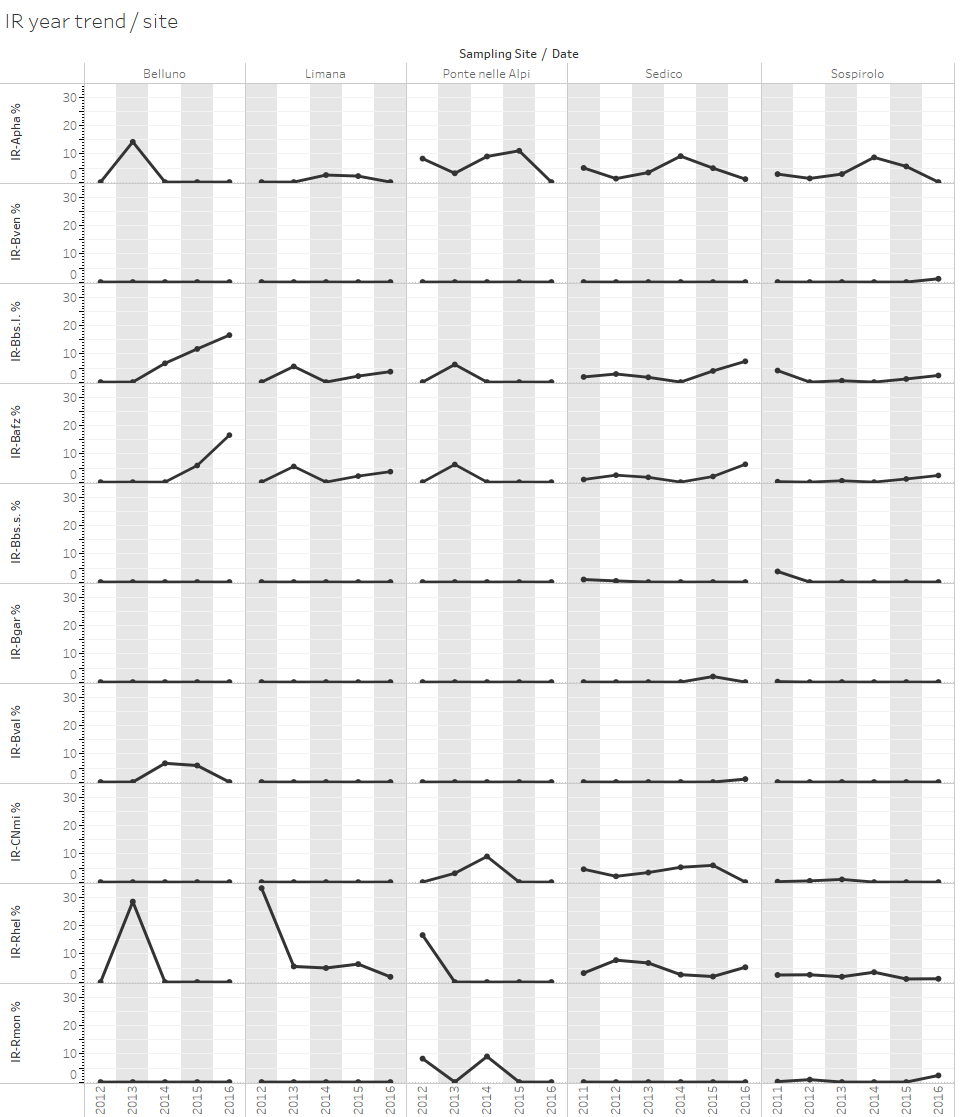

Supplement: Supplementary file 3 — Figure S2. Pattern of tick-borne pathogens found in Ixodes ricinus in the five permanent sites according to year of sampling. Abbreviations: Apha, Anaplasma pagocytophilum; Bbs.l., Borrelia burgdorferi (sensu lato) complex; Bafz, Borrelia afzelii; Bbs.s., Borrelia burgdorferi (sensu stricto); Bval, Borrelia valaisiana; CNmi, “Candidatus Neoehrlichia mikurensis”; Rhel, Richettsia helvetica; Rmon, Rickettsia monacensis. (TIFF 161 kb) [file 13071_2018_2726_MOESM3_ESM.tif]
